# Use of Matrix

**Published:** 1904-11

**Authors:** 


Use of Matrix.—We have all tried and know what results are obtained where a matrix is used, so far as this relates to making gold fillings. If there is a place in dentistry where there are contraindicted it is in connection with use of gold. The matrix and amalgam go hand in hand together, and there let the matter rest. In the fewest possible words, matrices have absolutely no place, nor are they useful adjuncts to employ where we are dealing with gold.—*International.*

